# Patients’ and caregivers’ perspectives on preparedness for and experience with chimeric antigen receptor T-cell therapy

**DOI:** 10.1093/oncolo/oyaf246

**Published:** 2025-08-05

**Authors:** Anna Barata, Bridget Coffey, Hermioni L Amonoo, Lara Traeger, Ashley M Nelson, Patrick Connor Johnson, Tejaswini M Dhwale, Kyle Karpinski, Heather S L Jim, Areej El-Jawahri

**Affiliations:** Department of Psychiatry, Massachusetts General Hospital, Boston, MA 02114, United States; Harvard Medical School, Boston, MA 02115, United States; Department of Psychiatry, Massachusetts General Hospital, Boston, MA 02114, United States; Harvard Medical School, Boston, MA 02115, United States; Department of Psychiatry, Brigham and Women’s Hospital, Boston, MA 02115, United States; Department of Psychosocial Oncology and Palliative Care, Dana-Farber Cancer Institute, Boston, MA 02215, United States; Department of Psychiatry, Massachusetts General Hospital, Boston, MA 02114, United States; Department of Psychiatry, Massachusetts General Hospital, Boston, MA 02114, United States; Harvard Medical School, Boston, MA 02115, United States; Harvard Medical School, Boston, MA 02115, United States; Department of Medicine, Division of Hematology & Oncology, Massachusetts General Hospital, Boston, MA 02114, United States; Harvard Medical School, Boston, MA 02115, United States; Department of Medicine, Division of Hematology & Oncology, Massachusetts General Hospital, Boston, MA 02114, United States; Department of Psychiatry, Massachusetts General Hospital, Boston, MA 02114, United States; Department of Health Outcomes and Behavior at the Moffitt Cancer Center, Tampa, FL 33612, United States; Harvard Medical School, Boston, MA 02115, United States; Department of Medicine, Division of Hematology & Oncology, Massachusetts General Hospital, Boston, MA 02114, United States

**Keywords:** chimeric antigen receptor T-cell therapy, symptom burden, quality of life, distress, caregiver

## Abstract

**Background:**

Chimeric antigen receptor-T (CAR-T) cell therapy has improved survival, yet its toxic profile may impact on survivors’ well-being. We examined patients’ and caregivers’ perspectives on the most common, severe, and distressing side effects across recovery (ie, first 60 days) and survivorship (ie, 60 days and onwards) and explored their perspectives on the education received to prepare for CAR-T.

**Materials and Methods:**

We conducted semi-structured interviews with patients and caregivers addressing (1) how they prepared for CAR-T, (2) the side effects experienced during the first-year post-treatment, and (3) how to best educate future patients and caregivers.

**Results:**

We included 19 patients and 12 caregivers. We identified 6 major themes. First, participants reported intense hope as well as stress and fear when presented with CAR-T, viewing it as a distinct and exceptional treatment. Second, following CAR-T infusion, some patients experienced intense symptoms (eg, fatigue, pain), while others were confused if not presenting with symptoms. Third, following discharge, patients experienced a myriad of ongoing symptoms, some of which were severe (eg, fatigue, cognitive symptoms, pain) and negatively impacted on their recovery. Fourth, some long-term survivors experienced ongoing symptoms but accepted this trade-off for being alive. Fifth, most patients and caregivers felt well prepared to cope with CAR-T, although some expressed the desire for more information on long-term side effects. Sixth, participants would recommend CAR-T to others while acknowledging the risk of experiencing side effects.

**Conclusions:**

Participants commonly reported significant symptoms throughout treatment and recovery. Additional education on long-term survivorship is needed.

Implications for Practice Patients undergoing CAR T-cell therapy experience a variety of side effects across recovery and survivorship that are distressing to both patients and caregivers. Results highlight the need to improve education on severe and long-term side effects to help better prepare patients and caregivers for treatment, as well as enhance supportive care efforts to optimize treatment outcomes.

## Introduction

Chimeric antigen receptor-T (CAR-T) cell therapy is leading to sustained durable responses in patients who would have otherwise poor prognoses. Nevertheless, CAR T-cell therapy is associated with several side effects that have the potential to impair patients’ recovery, including cytokine release syndrome (CRS), the immune effector cell-associated neurotoxicity syndrome, cytopenia, aplasia, neurologic and psychiatric disorders, and secondary malignancies among others.^1–3^

Despite its toxic profile, few studies include the patient and caregiver perspective. Evidence suggests that patients experience remarkable symptom burden prior to CAR-T^4–6^ and that symptoms peak around 1 week after infusion^7^^,^^8^ when up to 86% of patients experience moderate or severe symptoms, including pain, fatigue, dyspnea, and insomnia among others.^4^^,^^7^ While most of these symptoms are transient and return to the same levels as prior to treatment by day 90,^7–9^ up to 70% of patients diagnosed with lymphoma experience moderate-to-severe symptoms during the first 6 months following infusion.^4^ In fact, between 20% and 30% of patients with lymphoma and multiple myeloma report worsening physical functioning, pain, and fatigue at 6 months relative to prior to treatment.^6^^,^^10^ Similarly, perceived cognitive functioning may worsen beyond day 90^11^ to an extend that around 40% of patients with leukemia experienced cognitive difficulties at a median of 3 years post-infusion.^12^ While this initial work suggests that CAR-T survivors experience remarkable symptom burden, they evaluated the patient experience mostly in the context of clinical trials,^5^^,^^10^^,^^13^^,^^14^ included homogeneous samples of patients with a single diagnosis (eg, lymphoma, multiple myeloma),^5^^,^^6^^,^^10^ and treated with a single CAR-T product.^5–8^^,^  ^13^^,^^15^ Similarly, we also lack understanding on whether educational programs are meeting the patients’ and caregivers’ needs to prepare for CAR-T treatment and recovery.^16^

The aim of the current study was to examine patients’ and caregivers’ perspectives on the most common, severe, and distressing side effects of diverse CAR-T cell therapies as well as the impact that side effects had on patients’ functioning during recovery (ie, first 60 days) and survivorship (ie, from day 60 and onward). We also explored patients’ and caregivers’ perspectives on the education received to prepare for treatment.

## Materials and methods

### Study design

We conducted a qualitative study using semi-structured interviews to comprehensively explore patients’ and caregivers’ experiences when preparing for and undergoing CAR-T, along with their perspective on the education received to prepare for treatment. We used a qualitative approach to expand on existing findings at the time of study design,^17^ which were limited to physicians’ assessment as part of clinical trials^2^^,^^18^ and a few quantitative studies examining patient-reported side effects.^7^^,^^8^ Directed content analysis guided the study design, including interview guide development, data collection, and data coding and analysis, to allow us to explore specific pre-defined areas of content, including patient and caregiver experiences and needs related to CAR-T. The study was conducted at the Moffitt Cancer Center. We followed the Consolidated Criteria for Reporting Qualitative Research reporting guideline and checklist to document findings.^19^ The Advarra Institutional Review Board approved the study.

### Participants and procedures

Eligible patients were English- or Spanish-speaking adults (≥18 years), diagnosed with a hematological malignancy, treated with CAR-T, and had an ongoing disease response. Ineligible patients had an uncontrolled psychiatric or neurological disease that impaired the ability to provide consent based on their clinicians′ assessment. We used purposive sampling to ensure diversity in age (ie, < 40, 41-65, >65 years old), race, gender, and time since CAR-T (ie, 14– to 30 days, 31– to 90 days, 91– to 180 days, 181– to 360 days, over 360 days posttreatment). Patients identified an informal caregiver. Eligible caregivers were English- or Spanish-speaking adults (≥18 years), self-identified as a caregiver for a patient treated with CAR-T and able to provide consent. Participants (patients, caregivers) provided informed consent and received $20 remuneration for participating in the study.

A.B. and H.J. developed semi-structured interview guides for patients and caregivers based on relevant literature, clinical experiences with patients treated with CAR-T, and expertise in conducting qualitative studies in patients and caregivers treated with other forms of cellular therapy (eg, hematopoietic cell transplant).^20^^,^^21^ We refined the interview guides after the first interviews to improve clarity. Between November 2021 and March 2022, A.B., with no prior clinical relationship with participants, interviewed patients and caregivers separately via videoconference over a range of 12 to 64 minutes. We recorded and transcribed the interviews. Interviews conducted in Spanish were translated and transcribed to English for analyses.

On the day of the interview, participants self-reported their demographics. We collected data from the patients’ medical record. Variables gathered are described in Table 1.

**Table:** **Table 1.** Patients and caregivers’ characteristics.

	Patients (*n* = 19)	Caregivers (*n* = 12)
**Age, median (range)**	68 (46-88)	69 (50-76)
**Gender, *n* (%)**		
**Male**	9 (47.4)	6 (50)
**Female**	10 (52.6)	6 (50)
**Ethnicity, *n* (%)**		
**Hispanic or Latino**	6 (31.6)	4 (33.3)
**Not Hispanic or Latino**	13 (68.4)	8 (66.7)
**Race, *n* (%)**		
**White**	17 (89.5)	11 (91.7)
**Black or African American**	1 (5.3)	1 (8.3)
**More than one race**	1 (5.3)	
**Education, *n* (%)**		
**High school graduate**	3 (15.8)	2 (16.7)
**Partial college or specialized training**	5 (26.3)	4 (33.3)
**College or university graduate**	5 (26.3)	4 (33.3)
**Graduate professional training (graduate degree)**	6 (31.6)	2 (16.7)
**Marital status, *n* (%)**		
**Currently married**	14 (73.7)	11 (91.7)
**Divorced**	3 (15.8)	
**Widowed**	1 (5.3)	1 (8.3)
**Never married**	1 (5.3)	
**Current employment, *n* (%)**		
**Employed (full-time)**	2 (11.1)	3 (25)
**Disabled**	4 (22.2)	
**Retired**	12 (66.7)	9 (75)
**Household annual income, *n* (%)**		
**$10 000-$19 999**	1 (5.3)	1 (8.3)
**$20 000-$39 999**	3 (15.8)	3 (25)
**$40 000-$59 999**	3 (15.8)	1 (8.3)
**$60 000-$100 000**	4 (22.2)	1 (8.3)
**Greater than $100 000**	3 (15.8)	3 (25)
**Prefer not to answer**	4 (22.2)	3 (25)
**Diagnosis, *n* (%)**		
**Diffuse large B-cell lymphoma**	5 (26.3)	4 (33.3)
**Mantle cell lymphoma**	2 (10.5)	1 (8.3)
**Follicular lymphoma**	4 (21.1)	2 (16.7)
**Multiple myeloma**	8 (42.1)	5 (41.7)
**Number of prior lines of therapy, mean (range)**	5 (2-11)	
**Prior receipt of an autologous transplant, *n* (%)**		
**Yes**	10 (52.6)	
**No**	9 (47.4)	
**Prior receipt of an allogeneic transplant, *n* (%)**		
**No**	19 (100)	
**Months since CAR T-cell therapy infusion, median (range)**	3 (0-34)	3 (0-34)
**CAR T-cell therapy administration, *n* (%)**		
**Clinical trial**	4 (21.1)	
**Standard of care**	15 (78.9)	
**CAR T-cell therapy product infused, *n* (%)**		
**Axicabtagene ciloleucel**	7 (36.8)	6 (50)
**Tisagenlecleucel**	2 (10.5)	
**Idecabtagene vicleucel**	8 (42.2)	5 (41.7)
**Brexucabtagene autoleucel**	2 (10.5)	1 (8.3)
**Cytokine release syndrome, *n* (%)**		
**Yes**	17 (89)	
**No**	2 (11)	
**Peak grade of cytokine release syndrome, *n* (%)**		
**0**	2 (10.5)	
**1**	12 (63.2)	
**2**	5 (26.3)	
**Neurotoxicity, *n* (%)**		
**Yes**	6 (32)	
**No**	13 (68)	
**Peak grade of neurotoxicity, *n* (%)**		
**0**	13 (68.4)	
**1**	1 (5.3)	
**2**	2 (10.5)	
**3**	3 (15.8)	
**Admission at the intensive care unit, *n* (%)**		
**No**	19 (100)	
**Days of hospital stay, median (range)**	12 (6-32)	11 (6-32)

Abbreviation: CAR T, Chimeric antigen receptor-T.

### Analyses

We conducted directed content analyses to examine qualitative data^17^ to comprehensively understand the experience of receiving CAR-T. We first developed a coding schema to classify the data into different categories and identify main themes. Here, the term theme refers to the systematic identification of patterns.^22^ Specifically, A.B., H.L.A., and B.C. familiarized with the data by reviewing initial transcripts at regular weekly meetings. Based on these initial transcripts, we derived codes deductively (based on the interview guide and prior work) and then developed additional codes inductively based on the raw interview data. We continued applying the coding structure to subsequent transcripts to iteratively revised the structure and then applied the structure to the remaining transcripts.^19^ A.B. and B.C. coded participants’ interviews and met weekly to resolve discrepancies. We summarized code groups across patients and caregivers’ perspectives into broader themes and subthemes and extracted quotes to highlight each one. We compared themes reported by patients and caregivers to identify observations that converged, diverged, or complemented each other to report the comprehensive experience of receiving CAR-T. We achieved thematic saturation^23^ with a sample of 19 patients and 12 caregivers.

We conducted descriptive statistics to examine demographic and clinical variables, including medians and range for continuous variables and proportions for categorical variables. We conducted analyses with SPSS v21.

## Results

### Participants characteristics

Overall, 30 patients and 13 caregivers were eligible and approached for study participation. Among them, 19 patients (10 female, median age 68 years) and 12 caregivers (6 female, median age 68 years) consented. Participants’ characteristics are described in Table 1. Patients mostly received CAR-T as part of standard of care (15/19, 78.9%), and the product infused was Idecabtagene vicleucel (8/19, 42.2%), Axicabtagene ciloleucel (7/19, 36.8%), Tisagenlecleucel (2/19, 10.5%), and Brexucabtagene autoleucel (2/19, 10.5). Caregivers acted in this role for a median of 3 months (range: 0-34 months), 10 were spouses to the patient, one was the patient’s father and another the brother-in-law. A total of 8 interviews were conducted in Spanish (5 patients, 3 caregivers).

#### Summary of themes

We identified 6 major themes regarding the education received and the side effects experienced when undergoing CAR-T. Representative quotes are presented in Table 2. Overall, patients and caregivers reported similar perspectives. However, caregivers also noted some side effects not reported by patients, including changes in usual behavior, low mood, apathy, delirium, hallucinations, and personality changes.

**Table 2.** Participants’ representative quotes.

**1. *When presented with CAR-T, participants felt intense hope as well as stress and fear since they perceived it was a distinct and exceptional treatment*** 1.1. The first-time participants were presented with CAR-T “We were relieved that there was another option other than R-CHOP which was very stressful, shockingly stressful for both of us” (caregiver) “CAR-T as the latest, greatest and probably the last chance of getting good quality of life going forward” (caregiver) “I had concern because I’ve heard all kinds of stories.” (patient) 1.2. Search for additional information “We were very excited about it, and of course, we did go on the internet and stuff.” (patient) “I didn’t research anything. I didn’t research any information about CAR T-cells. Nothing. I said, “I don’t want to read anything. I am just going to listen to the doctors and that’s it” (patient) “My own deep dive into the literature frankly did not serve me well because a lot of the things that I was reading was kind of dire in terms of the prognosis and the treatments. So, I stopped looking at literature, attending remote conferences and trying to become an expert.” (patient) 1.3. Balancing the risks and benefits of CAR T-cell therapy “Since they had already told me that I had only six months left to live, I was going to get the therapy regardless of what they explained to me.” (patient) “Seeing all of the information of people who have managed to improve their health after CAR T-cells, encouraged me to face the risks. I didn’t have a problem doing so.” (patient)
***2. Following CAR-T infusion, some patients experienced intense symptoms, others were confused and feared not presenting with symptoms, and still others did not recall experiencing neurotoxicity.*** “I could hardly turn over in the bed [because of weakness]” (patient) “Not being able to maneuver like she used to was the side effect that worried me the most” (patient) “ve never had chills like that. I mean, on a scale 1 to 10, it was a 20” (patient) “(..) sometimes I’d want to say something, and I couldn’t find the words. So, I would try to find the right word and work on that because many times I switched one word for the other. (..) And I would say, “That’s not what I mean. Give me a second because I want to say something entirely different.” (patient) “I saw that little by little I was able to coordinate my movements better. It took me a few days. But I started coordinating my movements, and my writing started to improve (..). It wasn’t too easy, but I did walk every day. But coordinating my movements was the hardest part.” (patient) “She had no clue who she was. She couldn’t talk. She didn’t know what day it was. She didn’t know where she was. Nothing. (..). I’ve never seen anybody in that state. I was in law enforcement for 35 years and I’ve never seen anybody that was eyes open and didn’t know where they were. Nothing” (caregiver)
***3. Following discharge, patients experience a myriad of ongoing symptoms, some of which are severe and negatively impact on patientś recovery.*** “I had lost my appetite. I ate very little. We would celebrate when I ate three meals a day.” (patient) “I would get nervous over stupid things. I thought about things that were not important to me before and I would worry about them.” (patient) “I can’t write almost because my hands are very weak, and I feel a lot of pain all the time. I also feel a lot of pain in my feet. They also get numb.” (patient) “I would say that the pain has increased. The pain in my bones. As time went by, I did notice that I developed more generalized pain in my body and bones.” (patient)
***4. Some long-term survivors experience ongoing physical and psychological symptoms but accept them as a trade-off for being alive.*** “Absolutely nothing. She´s been the same person since day one other than being upset about her hair” (caregiver) “She still is suffering from I would say some form of being cold all the time when, you know, the house is quite warm” (caregiver) “Every now and then I feel like I wish I could get out and do a little more, but it’s not dampening too much.” (patient) “People will tell me stuff, and it’s stuff I’ve done with them, but I get a blank, I don’t remember it (..). It’s not there in that brain.” (patient) “It had a major effect on my brain. I feel confused. I forget things. I think it’s still a little bit like that. I feel that was because of that, the CAR T, but I can’t complain. I really am blessed.” (patient) “There are times when I feel a bit sad. I feel that my mood changes. I worry about it coming back. I want to erase it [the multiple myeloma] from here. I am working on that but it’s not easy for me. So, I am taking the medication they [clinicians] gave me after the therapy.” (patient) “On one hand I thought, great, okay, now my cancer is in remission but there is this raging pandemic and if I get inadvertently exposed I'm gonna die because that’s who was dying.” (patient)
***5. Most participants felt well prepared to deal with CAR-T, although some would like to know more information on long-term side effects*** 5.1. Preparation received for CAR-T “I feel like I was pretty well prepared, as much as I wanted to be.” (patient) “I had the information from the doctor and I pretty much knew what to expect. I mean not entirely but I had an idea that it was gonna be a difficult time in my life having CAR-T and following CAR-T.” (patient) “It would be good to know more about the effect it would have on your—the long-term effect on your immune system.” (patient) “I’d like to know if any patients have had neuropathy like I do that was not alleviated by CAR-T. I thought going in there was a possibility, maybe even a probability, that I would have pain loss. I’m not complaining because I’m alive.” (patient) “It’s very daunting to be immunocompromised and unable to be vaccinated during this pandemic. So, that wasn’t something that I had prepared for” (patient)
***6. Participants would recommend others to undergo CAR-T while acknowledging the risk of experiencing side effects.*** 6.1. Preparing other patients to receive CAR-T “I would encourage them. I have a friend I am encouraging. The reason why he hasn’t accessed this is financial. But I would tell them that this is one of the best ways to face this situation with more hope. That is what I would tell anyone.” (patient) “I think I would tell them to weigh their options carefully and to read. Don’t just take the pamphlets and everything home. But to read those carefully and to go into it with an open mind and stay positive.” (patient) “I would prepare them for side effects and for being discouraged about them such as experiencing difficulties coordinating, but seeing progress thereafter.” (patient) 6.2. Recommendations to manage side effects patients would give to patients when receiving CAR-T “To not have any expectations, take every day separately. Don’t use the word should, just do what you can and no more than that every day. You’ve got to rest, rest, you want to walk, walk, you want to eat, eat, you don’t, don’t worry, just be.” (patient) “Be patient because it’s gonna take a little while, so be patient. You’re gonna feel really weak but there will be a time when your life is gonna be much, much better.” (patient) “You have to take what’s advised, otherwise you’re just gonna be miserable through the whole thing and that’s all I can think of.” (patient)

Abbreviation: CAR T, Chimeric antigen receptor-T.

#### Theme 1: When presented with CAR-T, participants felt intense hope as well as stress and fear, as they viewed CAR-T as a distinct and exceptional treatment

When presented with CAR-T, some participants reported being excited and enthusiastic, while others felt “scared,” and “emotionally exhausted” when they learned about side effects and complications. One caregiver reported feeling stressed about having to conduct basic medical procedures (eg, cleaning the ports) but did not report this concern to the team, worrying that it could prevent their loved one from receiving CAR-T. Notably, most participants focused on the benefits of CAR-T rather than its risks arguing that “they had no choice” in order to live or that the possibility to prolong their life outweighed their concerns.

A large majority of participants sought additional information online or discussed treatment with non-CAR-T clinicians (eg, local oncologist, pharmacists). This information was beneficial for some participants, whereas others felt “frightened” and “terrified” when they learnt more about side effects and prognosis. Some other participants did not search additional information and trusted their clinicians to provide them with “the amount and kind of information” they needed, or did not want to read information that could have discouraged them. Still other patients mentioned that family and friends researched additional information and shared it with them which provided them with reassurance.

#### Theme 2: Following CAR-T infusion, some patients experienced intense symptoms, others were confused and feared being asymptomatic as a sign that CAR-T did not work and others did not recall experiencing neurotoxicity

Following CAR-T infusion, a few participants noted that the patient experienced no symptoms or “minimal fatigue,” which concerned some caregivers about CAR-T “not working.” In contrast, most patients experienced fatigue and weakness, which was severe for some, delayed recovery and discharge, and were distressing for their caregivers (“She was very fatigued and not able to really care for herself without assistance”). For most patients, fatigue and weakness slowly improved during hospitalization.

A large proportion of patients experienced CRS, or a myriad of symptoms such as fever, chills, sweat, shivers, blood pressure fluctuations, hypoxia, feeling cold, “cognitive reactions,” anorexia, and headache. These symptoms were severe and distressing for some participants (“I remember that I kept talking to this kid that I thought was in the room but he wasn′t.(.) My brain was the worst, thinking and doing things weird”) but not for others (“I thought it [the CRS] was something benign”). Most participants reported that the fever, chills, and low blood pressure resolved during hospitalization.

Some patients experienced neurotoxicity or symptoms such as aphasia, confusion, dysgraphia, difficulties focusing, difficulties with coordination and memory, delirium, hallucinations, “cognitive changes,” or “brain necrosis.” One patient and several caregivers reported changes in the patient’s usual behavior. Most patients did not recall neurological symptoms but were told by clinicians and their caregivers (“The worst part about the CAR-T in the hospital was knowing that something had happened and I didn′t recall it”). Other patients were aware of experiencing aphasia, confusion, dysgraphia, and difficulties with coordination, and memory gaps and described them as “the hardest,” “the worst,” “when (you are) the most discouraged.” Caregivers reported feeling “disturbed” and “shocked” when patients did not recognize who, when, and where they were, nor were they able to write. Some participants reported that the neurological symptoms resolved within hours or days.

Several patients experienced pain, which was generalized for some and localized for others, but interfered with patients’ recovery (“The pain that developed in my right knee (.) it was excruciating pain. To the point where I couldn′t walk”) and concerned them about cancer recurrence. One patient described that pain resolved at time of discharge. Some patients reported experiencing symptoms of anxiety due to concerns about treatment failure and side effects. Caregivers reported that patients experienced mood swings, low mood, and lack of motivation. Additionally, a few participants also reported that the patient experienced severe insomnia, hypersomnia, infections, neutropenia, difficulties with vision, hypothyroidism, neuropathy, frequent and painful urination, and financial toxicity.

#### Theme 3: Following discharge, patients experience a myriad of ongoing symptoms, some of which are severe and negatively impact on patients′ recovery

Several patients experienced ongoing symptoms following discharge. Most patients experienced fatigue and weakness, which were severe for some participants (“I was very weak, it was a luxury for me to go out on walks”) despite most expressed ongoing improvements during this time.

More patients reported experiencing cognitive symptoms relative to hospitalization, including difficulties with memory (“He couldn′t remember how to operate an iPhone”), difficulties learning new information, confusion, drowsiness, difficulties finding the correct word or naming an object, and unsteady coordination and balance. Some patients reported that some cognitive symptoms improved at this time (eg, confusion, difficulties with coordination and balance, tremors).

Several other patients experienced persistent gastro-intestinal symptoms such as altered smell and taste, nausea, lack of appetite, and diarrhea which was severe for some patients and stressful for their caregivers. Participants reported that gastro-intestinal symptoms generally improved during this time.

Several patients reported mood swings and symptoms of anxiety, including irritability and worry, whereas several caregivers noted the patients experienced low mood and lack of motivation. Additional symptoms that persisted in few participants during this time included generalized severe pain, hypersomnia, neutropenia, shortness of breath, difficulties with vision, and anemia with some of these symptoms bothering both patients and caregivers.

#### Theme 4: Some long-term survivors experience ongoing physical and psychological symptoms but accept them as a trade-off for being alive

Participants reported that the severe side effects that most often impaired long-term recovery were fatigue and weakness (“So he would go and wash dishes and then he would have to sit down and rest and then go back and finish washing the dishes”), immunodeficiency, difficulties concentrating and remembering information (“My memory is terrible. That′s my new normal”), and psychosocial distress (“I thought I felt something [tumor] one day a couple of months after my CAR-T, and it undermined all my newfound perspective and I lost it. I was in such a panic that my wife became panicked”). Participants reported that these side effects impaired their ability to walk unaided, drive, receive vaccinations, resume their social lives and hobbies, and made them feel insecure and uncomfortable when surrounded by others. Additionally, participants reported lingering difficulties with coordination, pain, irritability, and altered sleep pattern (insomnia, hypersomnia). Caregivers also noted changes in patients’ personality with patients being more isolated. Despite this, nearly all patients reported that they would still have gone through CAR-T given all they knew about it now.

Several participants reported that the patient was feeling better than expected considering the risk of side effects and the poor prognosis prior to CAR-T. Some patients experienced ongoing side effects but expected these trade-offs based on the education received from clinicians. Still other participants reported having no expectations but were instead focused on the patient being alive while hoping for a continued recovery. In contrast, some participants reported feeling worse than expected and were surprised by the ongoing and “daunting” side effects and the long and “frustrating” recovery.

#### Theme 5: Most participants felt well prepared to deal with CAR-T, although some would like to know more information on long-term side effects

Most participants were satisfied with the education received prior to CAR-T. There were few suggestions to improve education about hospitalization, and most focused on long-term side effects and expected recovery time. A few caregivers wanted to know more about severe neurotoxicity. Some participants preferred to receive additional education prior to CAR-T, while few others mentioned ongoing reminders throughout the CAR-T trajectory or at 1-year post-treatment. Similarly, most participants preferred to receive additional information from clinicians, whereas others also wanted to hear it from patients and caregivers. Additional suggestions included providing future patients and caregivers with access to online resources they could trust, creating a “a library of patient testimonials that people could view at their own pace” or a brochure condensing all the information relative to the hospitalization and the timeline of expected check-ups following discharge.

#### Theme 6: Participants would recommend others to undergo CAR-T while acknowledging the risk of experiencing side effects

Some participants would encourage others to receive CAR-T based on the potential to control their disease, while also mentioning the risk of experiencing side effects. Few participants would share their experience with others. In contrast, other participants would not “discourage” anyone but would prepare them to expect side effects and a lengthy recovery. Caregivers would prepare future caregivers for a long and “hard” treatment, that can lead to significant out-of-pocket expenses and requires caregivers not to leave the hospital while the patient is hospitalized. Caregivers described their role as physically and psychologically “hard.” Caring for the patient at home after discharge was “stressful” and the “worst,” although some also expressed being grateful and blessed for taking care for their loved one. Similarly, several patients expressed gratitude for the opportunity to receive CAR-T, for the support they received from their caregivers and clinicians, and for the altruism of other patients who volunteered in the research that led to CAR-T approval.

## Discussion

Chimeric antigen receptor-T has provided hope to patients and caregivers confronted with relapsed and recurrent diseases, while also causing a variety of side effects that interfere with patients’ functioning and are distressing to both patients and caregivers.

Patients experience a variety of symptoms during the first-year post-infusion, and long-term survivorship is characterized in some patients by fatigue, weakness, cognitive symptoms, pain, immunodeficiency, and psychosocial distress. Although most participants reported being well-prepared to cope with CAR-T, some were shocked and “discouraged” when faced with severe side effects, and still others would like to know more information on long-term side effects. Prior work noted that when clinicians present CAR-T, they tend to focus on the manufacturing process, the possibility of prolonged disease control, and strategies to address risks and complications but may overlook side effects, life-threatening complications, and treatment failure.^24^ Additional educational efforts should focus on presenting CAR-T comprehensively to better prepare patients and caregivers. These efforts should include the experience of prior patients and caregivers as well as accommodate the different strategies that patients and caregivers have to cope with threatening information (ie, some actively seek additional information, others want to fill in gaps, and still others avoid threatening information). A Web page or a digital health application with a menu of topics and testimonials could allow future patients and caregivers to select the information and experiences they are interested in reviewing. Similarly, additional research should collect and publish patient-reported data in a way that is easy for patients and caregivers to understand so they can better prepare for treatment.

Overall, patients experienced substantial symptom during hospitalization, recovery and still others during long-term survivorship, but a majority accepted this trade off in order to be alive. In fact, almost all patients would recommend CAR-T to others and would go through treatment again given their experience. Nevertheless, prior work noted that treatment-regret may be common among patients who relapse after cellular therapy.^25^ Additional educational efforts are needed to improve treatment outcomes. For example, an educational video to prepare patients for CAR-T increased patients’ self-efficacy and treatment-decision satisfaction relative to participants who received usual care.^26^

Notably, our findings also have practical policy implications. The substantial symptom burden and quality-of-life impairments observed underscore the need to involve palliative care clinicians in the care of these patients and refer patients in need to additional supportive care resources, including mental health providers or rehabilitation. Similarly, the remarkable symptom burden may increase the overall treatment cost due to greater healthcare utilization (eg, prolonged hospitalizations, emergency department visits, supportive care resources) and medication (eg, pain management, insomnia). Likewise, policy makers should also consider the potential of symptom burden to delay patients’ and caregivers’ return to work and therefore increasing patients’ financial toxicity. Overall, these findings provide important context to plan for supportive care resources for the CAR-T population. This is critical considering that CAR-T is increasingly administered,^27^ with projections estimating that in the next 5-10 years, 2 million patients can be considered candidates to receive CAR-T.^28^

Our study had also limitations that should be noted. First, although a large percentage of the sample were Spanish-speaking, the sample was comprised of mainly White, married, and retired participants. While we reached thematic saturation with this sample, participants with different demographic characteristics may have reported different experiences. Second, we included patients and caregivers with ongoing disease response and did not capture the symptom burden and education needs of those whose disease did not respond. Third, we conducted directed content analyses which allowed us to describe the experience of patients and caregivers when preparing for and undergoing CAR-T, whereas the interview guide focus and resulting data limited the possibility to conduct more interpretative analyses. Finally, we conducted the study at a single large academic medical center in the United States and thus the experiences herein reported may not generalize to different settings. Additional studies that capture the hallmark side effects of CAR-T and the overall symptom burden are clearly needed to better comprehend its impact on patients and caregivers.

The study findings highlight that CAR-T is associated with symptom burden during hospitalization and recovery and still for some during long-term survivorship. Additional educational and supportive care efforts are needed to better prepare patients and caregivers for CAR-T throughout treatment.

## Data Availability

The data underlying this article will be shared on a reasonable request to the corresponding author. For original data, please contact abarata@mgh.harvard.edu
